# Avian encephalomyelitis virus in backyard chickens

**DOI:** 10.14202/vetworld.2023.1866-1870

**Published:** 2023-09-17

**Authors:** Abdullah I. A. Al-Mubarak, Jamal Hussen, Mahmoud Kandeel, Anwar A. G. Al-Kubati, Baraa Falemban, Maged Gomaa Hemida

**Affiliations:** 1 Department of Microbiology, College of Veterinary Medicine, King Faisal University, Saudi Arabia; 2 Department of Biomedical Sciences, College of Veterinary Medicine, King Faisal University, Al-Hofuf, Saudi Arabia; 3 Department of Pharmacology, Faculty of Veterinary Medicine, Kafr Elsheikh University, Kafr Elsheikh, Egypt; 4 Department of Veterinary Medicine, Faculty of Agriculture and Veterinary Medicine, Thamar University, Thamar, Yemen; 5 Department of Veterinary Biomedical Sciences, College of Veterinary Medicine, Long Island University, USA

**Keywords:** antibody, avian encephalomyelitis virus, backyard, chicken, encephalomyelitis virus, enzyme-linked immunosorbent assay, seroprevalence, the prevalence, wild birds

## Abstract

**Background and Aim:**

Avian viral diseases usually cause high economic losses because of high morbidity and mortality and poor growth. The rearing of chickens in backyards could have an important role in the spread of certain diseases, particularly those of viral origin. Infected birds might be prone to many viral infections for several reasons, including a lack of vaccination programs, the mixing of different bird species in the same location, and the close interactions of these birds with wild and migratory birds carrying various pathogens. This study aimed to conduct serological surveillance of avian encephalomyelitis virus (AEV) in some backyard chickens in the eastern region of Saudi Arabia.

**Materials and Methods:**

Serum samples (n = 368) were collected from domestic chickens reared in 10 backyards in the Eastern Province of Saudi Arabia. None of the domestic birds in these 10 backyards were vaccinated against the virus. In addition, 78 serum samples were collected from free-ranging birds belonging to Columbidae, such as pigeons and doves, in common areas near the domestic backyards. We tested these sera for specific antibodies against AEV.

**Results:**

Our results revealed seroconversion to AEV among the examined chickens (14.6%). None of the tested pigeons and doves displayed seroconversion to AEV.

**Conclusion:**

Seroconversion of these non-vaccinated birds against AEV was suggestive of a recent natural infection by this virus. Further studies with a large number of birds are required to molecularly characterize the circulating strains of this virus in this area.

## Introduction

Backyard bird breeding is commonly practiced in some countries. In these backyards, several species of birds, chickens, geese, quails, and turkeys are usually kept together in a small breeding area with shared food and water sources. Meanwhile, birds of various ages can be found in these backyards. In most cases, these backyard birds are not vaccinated against the most common diseases of birds, particularly viral diseases. Furthermore, some wild birds can enter these backyards for food and water. This environment permits viral disease transmission between domestic and wild birds [1]. Several outbreaks of different virus diseases affecting various species of birds have been reported in backyards, such as avian influenza, Newcastle diseases, avian encephalomyelitis, Marek’s diseases, and fowlpox virus [1–6]. We are investigating the exposure history of backyard birds to avian encephalomyelitis virus (AEV) in the eastern region of Saudi Arabia.

Avian encephalomyelitis virus is a highly contagious virus in chickens. It is called an epidemic tremor-inducing virus because it infects the central nervous systems of young chickens, leading to nervous manifestations such as ataxia, tremors, paresis, and ultimately paralysis [7]. In some cases, AEV infection in chickens is associated with diarrhea, poor feed conversion rates, and stunted growth, contributing to malabsorption syndrome [8]. Avian encephalomyelitis virus is usually transmitted through horizontal and vertical pathways, particularly the fecal-oral route, through the ingestion of contaminated food and water [9]. Avian encephalomyelitis virus has a wide host range among poultry species. This virus can infect chickens, pheasants, quails, and turkeys. Avian encephalomyelitis virus infection in turkeys is usually mild [10]. Avian encephalomyelitis virus is a positive-sense RNA virus belonging to the Picornaviridae family and *Enterovirus* genus [9]. Avian encephalomyelitis virus also infects some wild birds and pheasants [11, 12]. Several approaches have been applied to diagnose AEV infection in chickens, such as virus isolation in chicken embryos. The most noticeable effect of the virus on embryonated eggs is paralysis of the embryos [13]. Other laboratory techniques used to diagnose AEV in chickens include real-time polymerase chain reaction using primers targeting the VP2 region of the viral genome [14]. Several serological surveillance studies revealed seroconversion to AEV in chickens in Nigeria, China, and Southern Mozambique [15–17]. Interestingly, the prevalence of AEV in the Gulf region and Middle East is unknown. Meanwhile, the roles of migratory and wild birds in AEV transmission among domestic chickens are not well studied in this area.

This study examined the exposure history of backyard chickens experiencing respiratory and enteric syndromes outbreaks. In addition, the seroconversion of wild and free-roaming birds living near these backyards was investigated.

## Materials and Methods

### Ethical approval

All handling and chicken experiments carried out in this study were conducted as per the instructions of the Animal Ethics protocols and the National Committee of Bioethics, King Abdul-Aziz City of Science and Technology (KACST), Royal Decree No. M/59. The chicken experiments and protocols were reviewed and approved by the animal ethics committee of the Deanship of Scientific Research, King Faisal University, Saudi Arabia (Approval No: KFU-REC/2020-12-35). All the necessary paperwork for sample collections was obtained.

### Study period and location

**Figure-1:**
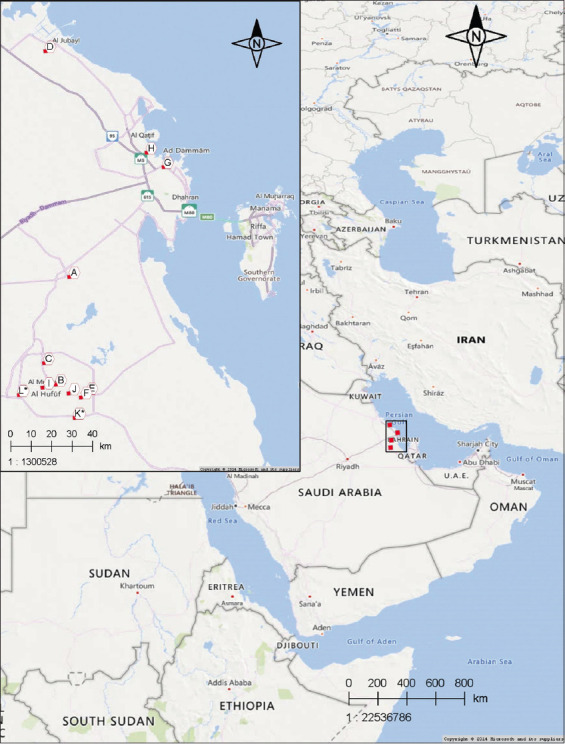
Map showing the backyard chickens’ geographical locations and the wild birds’ hunting spots [Source: www.maps.google.com].

Samples were collected from November 2018 to April 2019. The samples were processed from July to September 2021. The geographic locations of the sample collection are marked on the map in Figure-1. The samples were processed at Virology Laboratory, College of Veterinary Medicine, King Faisal University, KSA.

### Sample collection from wild free-range birds

**Figure-2:**
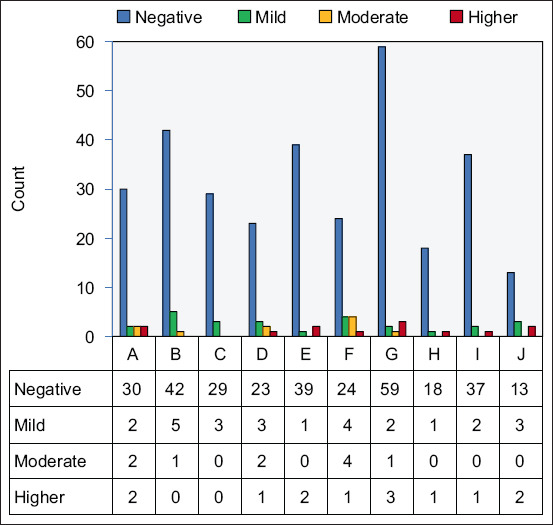
Histogram illustrates the scoring of the various birds in each outbreak in the backyard chickens based on the level of the avian encephalomyelitis virus detected antibodies in their sera.

Professional hunters captured free-range birds such as pigeons, sparrows, seagulls, and doves. Simply, they fixed nets in locations close to the backyards of chickens under study. The captured birds were collected early in the morning and transferred to the laboratory daily. The total number of samples per location is shown in Figure-2.

### Sample collection from chickens and wild free-range animals

Blood specimens were obtained from the wing veins of domestic and wild birds as previously described [18–20]. Serum was obtained from the blood samples and stored at −20°C for serologic testing.

### Indirect enzyme-linked immunosorbent assay (ELISA) for AEV

We used the ID Screen^®^ AEV-Indirect (ID.Vet-catalog number: AEVS-5P, 310 rue Louis Pasteur – 34790 Grabels, France) to test the collected serum samples for antibodies against AEV. Enzyme-linked immunosorbent assay was performed per the instructions of the kit. A 1:500 dilution was prepared from each serum sample using the kit dilution buffer. Microtiter plates precoated with AEV antigen were filled with 100 μL of serum samples, negative, or positive controls. Then, the plates were incubated for 30 min at 21°C, followed by five washes with wash buffer. The antichicken IgG antibody conjugate was added to the wells, followed by incubation for 30 min at 21°C. After three washes, the plates were filled with 100 μL of 3,3’,5,5’-tetramethylbenzidine substrate solution, followed by incubation for 30 min at RT in the dark. Finally, the reaction was stopped by adding a stop solution, and the color density was measured at 450 nm using an ELISA reader. The test was considered valid if the ratio between the optical densities (ODs) of the positive and negative controls exceeded 3. A scoring system was developed for the level of antibodies in the sera of each bird. The sample-to-positive control ratio and the antibody titer was calculated. The cutoff was 853. Samples were considered negative when OD < 853, whereas samples were considered positive as follows: (+), OD = 854–1706; (++), OD = 1707–2559; and (+++), OD > 2559.

### Statistical analysis

We used the Kruskal–Wallis test to compare the prevalence of AEV among the tested locations as previously described [20, 21].

## Results

### Detection of antibodies against AEV in the sera of domestic backyard chickens

We tested 368 chickens from 10 locations in the eastern region of Saudi Arabia for the presence of AEV antibodies (Table-1 and Figure-1). The overall seroprevalence of AEV among the tested chickens was 14.6%. The highest seroprevalence was detected in backyard-J, whereas the lowest prevalence was recorded in backyard-C (Table-1, Figure-2). Scores of (+), (++), and (+++) were identified for 26, 14, and 13 chickens, respectively.

### Detection of antibodies against AEV in the sera of free-roaming birds in the area of study

We captured 78 birds at two locations, as presented in Figure-1. We tested the sera of these birds for antibodies against AEV. Our data illustrated that none of the tested wild birds seroconverted to AEV (Figure-2).

## Discussion

The exposure of domestic birds, particularly chickens in backyards, to exotic and wild birds could be linked to the transmission of viral pathogens, including AEV [22], and the transmission is bidirectional. Backyard breeding is growing in many countries, and it is considered a major risk factor for the emergence of pathogens because of insufficient biosecurity standards [22]. Thus, active surveillance strategies are highly recommended for monitoring the emergence of any new pathogen and any outbreaks of known pathogens among backyard birds. Previous studies revealed that backyard chickens might play important roles in transmitting other viral diseases, such as avian influenza virus A, H5N8, and infectious bronchitis virus [4, 23–27].

Similarly, we investigated several outbreaks in backyard chickens exhibiting respiratory and nervous signs, particularly tremors in the head and neck, and enteric syndrome in Eastern Saudi Arabia. Our goal was to test these chickens for the presence of AEV in their sera. Our results revealed seroconversion to AEV in 14.6% of the tested birds. We noticed huge variations in the AEV antibody level in these sera of the birds. We developed our scoring system to classify seropositivity based on the level of antibodies against AEV. Based on the established cutoff for positivity (OD = 853), the antibody levels in the birds’ sera varied from extremely low to moderate or high. This heterogeneity in the level of antibodies against AEV in the sera of the tested birds could have several explanations. First, the variations could be related to individual differences in genetic susceptibility and resistance to certain pathogens. Second, some birds might have experienced multiple cycles of AEV infection, particularly those in the (+++) group. The birds were not vaccinated against AEV, and they shared a management system. Meanwhile, these backyards were located in small towns or villages distant from commercial large-scale poultry production or farms. This eliminated the possibility that viral infection was linked to commercial poultry farms. Although none of the tested free-range and wild birds tested positive for AEV antibodies, the testing of large numbers of birds and other species is needed to fully address the roles of these birds in the sustainability and transmission of AEV to domestic birds.

The previous reports recorded a much higher seroprevalence of AEV than our study. The seroprevalence was 79% in a Brazilian study [28], 45%–91% in Bangladesh [29], 79.35% in the Tamil Nandu state of India [30], and 57.1% in Sudan [31]. Reports on the incidence of AEV described the recent detection of AEV in several countries. In 2021, AEV was first reported in Bangladesh [29]. The detected low incidence of AEV in this study might be associated with the low density of chicken flocks in the collected sample areas. It has been suggested that seropositivity is associated with larger chicken flocks rather than smaller flocks [29]. The high prevalence in some areas and the first reporting of AEV suggest the potential spread of the virus in new territories and the emergence of an expanded host range, as well as the potential for viral epidemics.

## Conclusion

The presence of antibodies against AEV in the sera of unvaccinated domestic chickens strongly suggests their exposure to the virus. Further studies are needed to explore the mode of transmission and to molecularly characterize the circulating strains of AEV in these birds.

## Authors’ Contributions

AIAA and MGH: Conceived the idea. AIAA: Funding and project management. AIAA, JH, MK, AAGA, BF, and MGH: Sample collection, laboratory testing, and data interpretations; wrote the manuscript. All authors have read, reviewed, and approved the final manuscript.

## Data Availability

The supplementary data can be available from the corresponding author on a request.
